# Pregnant women affected by thalassemia major: a controlled study of traits and personality

**Published:** 2010

**Authors:** Giuseppina Messina, Elisa Colombo, Elena Cassinerio, Claudia Cesaretti, Alessia Marcon, Laura Zanaboni, Marina Baldini, Maria Domenica Cappellini

**Affiliations:** a Hereditary Anemia Center, Department of Internal Medicine, Policlinico Foundation IRCCS, University of Milan, Milan, Italy; b Unit of Psychiatry, Policlinico Foundation IRCCS, University of Milan, Milan, Italy; c Unit of Endocrinology, Policlinico Foundation IRCCS, University of Milan, Milan, Italy

**Keywords:** Personality, Pregnancy, Thalassemia Major

## Abstract

**BACKGROUND:**

The reproductive and sexual health issues concerning persons affected by thalassemia major are complex. The study was planned to investigate the psychological attitudes and expectations in a group of thalassemic pregnant women attending hospital for regular blood transfusion.

**METHODS:**

This is a preliminary cross-sectional study involving 20 consecutive thalassemic patients and a control group of 42 healthy pregnant volunteers. The personality structure was evaluated by Rorschach’s test and the presence of psychic symptoms by SCL-90-R and STAI.

**RESULTS:**

Narcissism and sexual traumas are significantly higher in thalassemic women with respects to the control group. Also the percent of anxiety and depression observed with the SCL-90-R was significantly higher than in control group (45% vs. 3%, p < 0.001, mean and SD values are 1.65 ± 0.15 vs. 0.43 ± 0.18 for anxiety; 55% vs. 12%, p < 0.001, mean and SD values are 1.76 ± 0.18 vs. 0.85 ± 0.25 for depression). The score observed with the STAI shows that the trait of anxiety differed between thalassemic pregnant women and the control group, even though the score values aren’t pathologic in neither group (87% vs. 42%, p < 0.05, mean and SD values are 33 ± 0.8 vs. 22 ± 0.2).

**CONCLUSIONS:**

This study addresses the need for developing, implementing and evaluating proper psychological support for thalassemic pregnant patients. Moreover, psychological screening and support prior to, during and following pregnancy would be indicated.

Due to the medical advancements in the fields of artificial insemination, of obstetrics and foetal and neonatal monitoring techniques, today it’s possible for a woman suffering from beta-thalassemia to have a successful pregnancy. Pregnancy-related problems affecting beta-thalassemic women are here discussed, emphasizing the connection between pregnancy management and gestation successful outcome.^1^,^2^ The patient affected by this disease is at high risk of reproductive disorders and associated psychosocial distress because of the chronic nature of the condition, the complexity of its treatment, and the well-documented complications of hypogonadotrophic hypogonadism; like arrested growth, delayed or absent development of sexual organs, infertility, and osteoporosis.^3^,^4^ In the past, children with homozygous thalas-semia rarely survived longer than adolescence^5^ and healthy sexual life was considered irrelevant in this group of patients. Improved medical treatments introduced in the late 1970s, which consists in regular transfusions of optimum red blood cell and almost daily subcutaneous iron chelation therapy, has significantly reduced the mortality rate among these patients and raised life expectancy from adolescence to midlife.^6^ As a result patient care has now expanded in order to encourage these patients to look for vocational, social, sexual, and reproductive goals which their peers without thalassemia normally can achieve in life.^7^,^8^ There is not much literature on the subject. Most of the published studies investigate the hypothalamic pituitary-gonad axis of these patients only from an endocrine perspective.^9^–^11^ Some studies examine the reproductive com plications that persons suffering from homozygous thalassemic may encounter; focusing on how these people perceive reproductive health within the frame of their disease and whether having thalassemia influences their reproductive behaviour.^12^–^16^ Despite an increasing number of thalassemic women which decide to have a baby, at present there aren’t studies which evaluates the pregnancy-related psychological conditions. Following recent discoveries in the psychology of thalassemic patients^17^,^18^ a preliminary study was performed to investigate the psychological profile of thalassemic pregnant women, trying to make a comparison between psychological conditions in women affected by the disease and healthy women, focusing on major personality traits of both groups.

## Methods

The study was conducted between June 2005 and December 2008. Twenty consecutive thalassemic patients (average age: 32 years, range: 24-41), who were admitted at Hereditary Anaemia Center of the Department of Intern Medicine of the University of Milan, Policlinico Foundation IRCCS, were recruited. During June 2005 and December 2008 all thalassemic pregnant women were invited to participate in the study. The response rate was 100%. The study was approved by the Regional Ethics Committee, the inclusion criteria were being pregnant and affected by thalassemia major who followed regular blood-transfusion therapy. The study was explained to each person and they all signed an informed consent. The patients were assessed with two interviews: the first investigated the patient’s personal history and collected her anamnesis in order to understand the reasons beneath the decision to have a baby. During the second one the tests for psychological profiling were administered to the patients. The control group consisted of 42 age-matched healthy pregnant volunteers. The characteristics of the patients are reported in table 1.

**Table 1:** Characteristics of 20 talassemic pregnant women and of 42 healthy pregnant volunteers

	Patient’s Group	Control Group
Characteristics	n = 20	n = 42
Median Age (years)	32 (range: 24-41)	30 (range: 28-40)
Married or Cohabiting	17	25
Number of Married Years, Mean (SE)	8 (3,2)	7 (1,5)
Education Middle School	12	29
High School	8	13

### 

#### Statistical Analysis Description

Data were statistically evaluated with the Chi square test and the Student’s t test adjusted with a correction factor, as appropriate. The results were considered to be statistically significant by p < 0.05; α and β were 5% and 95% respectively. Proportions were compared by using the Chi square test, whereas the means by using the Student’s t test. All authors had full access to the data and take responsibility for its integrity and accuracy of the analysis. All authors have read and agreed to the manuscript as written.

#### Interviews

The thalassemic women who accepted the psychological investigation were submitted to the first face-to-face interview not structured of introduction and anamnesis of the duration of about one hour. By the mean of this interview information was obtained about social relationships, job and motivations and expectations related to pregnancy, previous organic diseases, family, sexuality and significant events like traumas and affective losses. The second psychological analysis of the duration of about two hours was planned to proceed with psychic profiling of the thalassemic patients; the Rorschach’s test was chosen because it can’t be influenced by the rational consciousness of the patient,^19^ the Symptom-check-list 90 revised and the STAI were chosen because they are short, they are validated in the local context, tools translated into local language and they are appropriate tests for patients with chronic disease.^20^,^21^ As far as test psychometric properties are concerned, the Rorschach’s test is the projective examination, whereas the other tests are consisting of self-reporting inventory.^22^,^23^ The Cronbach’s alpha value is 0.86 for STAI and 0.82 for SCL-90-R. The interviews have been conducted by a psychologist (GM).

#### The Rorschach’s Test

The Rorschach’s test is a projective personality assessment based on the client’s reactions to a series of 10 inkblot pictures. It is the most widely used projective psychological test. The Rorschach’s test is used in the assessment of personality structure and identification of emotional troubles and mental disorders. It is based on the principle that when patients are stimulated with neutral and ambiguous images, they will respond projecting their own personality in order to interpret them, thus revealing a variety of unconscious conflicts and motivations. The Rorschach’s test is used to elicit information about the structure and dynamics of an individual functioning personality. The test provides information about a person’s thought processes, perceptions, motivations and attitude towards the environment and it can detect internal and external pressures and conflicts as well as illogical or psychotic thought patterns. The Rorschach’s test is administered using 10 cards, each containing a complicated inkblot pattern; five cards in black and grey, two in black and red, and three in various pastel colours. Patients look at the cards one at a time and describe what they see in each inkblot. They are instructed to look at the shape, the colour and the shading of the inkblots. After the person has viewed all 10 cards, the examiner usually goes back over the responses for additional information. The person may be asked to clarify some responses or to describe which features of each inkblot prompted the responses. There isn’t a right response to an inkblot, although there are common responses to some of the cards. Despite several critics the Rorschach’s test is considered an adequate instrument in psychological diagnosis and in the study of unconscious personality profile.

#### The Symptom-Check-List 90 Revised (SCL-90-R)

The SCL-90-R is a multidimensional self-reporting symptom inventory consisting of 90 items each measured on a 5-point scale of distress from “not at all = 0” to “extremely = 4”. The SCL-90-R quantifies psychopathology in terms of nine primary symptom constructs: Somatisation (SOM), Obsessive-Compulsive Disorder (DOC), Interpersonal Sensitivity (IS), Depression (DEP), Anxiety (ANX), Paranoid Ideation (PAR), Hostility (HOS), Phobic Anxiety (PHOB) and Psychotics (PSY). Moreover, 3 global indices of distress, the global severity index (GSI), the positive syndrome distress index (PSDI) and the positive symptom total (PST), reflect respectively the current level or depth of the disorder, the response style (“growing” or “lessening”) and the number of symptoms reported as positive.

#### State and Trait Anxiety Inventory (STAI)

The State and Trait Anxiety Inventory (STAI) is the definitive instrument for measuring anxiety in adults. The STAI clearly differentiates between the temporary condition of “state anxiety” and the more general and longstanding quality of “trait anxiety”. The STAI is a 20-item self-report form that is a reliable and well-validated measure of acute anxiety. The essential qualities evaluated by the STAI scale are feelings of apprehension, tension, nervousness, and worry. Scores on the STAI scale in crease in response to physical danger and psychological stress, and decrease as a result of relaxation training. On the STAI scale, consistent with the trait anxiety construct, psychoneurotic and depressed patients generally have high scores. Average scores on the STAI vary with age and gender. If the score is more than 40 the trait of anxiety is pathologic; if it is less than 40, the trait of anxiety is normal.^24^

## Results

**Figure 1:**
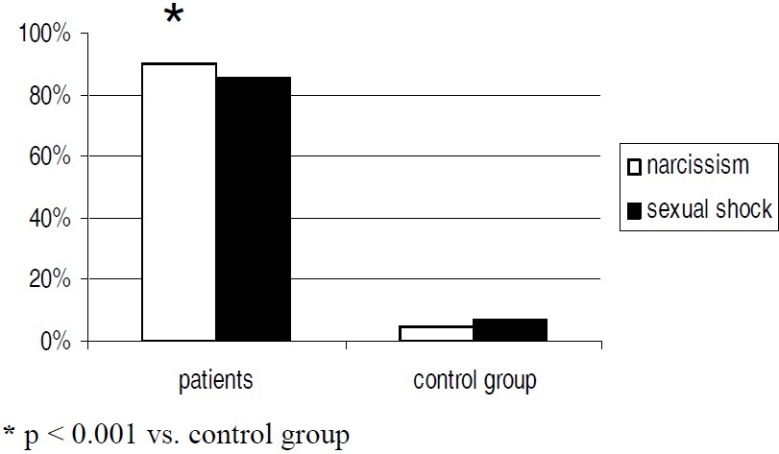
Response to Rorschach’s test in thalassemic women and in healthy control

The responses to the Rorschach’s test are reported in figure 1. The principal personality traits which appear from the test are narcissism and sexual traumas. Narcissism emerges from the high number of Rorschach’s responses in which the patient saw reflexes or mirrored images. Rorschach’s test is composed by a Sexuality card (number VI); thalassemic women often showed a rejection in front of this card or else the response time increased considerably and then the patient said that she didn’t see anything at all.

Narcissism and sexual traumas are significantly higher in thalassemic women with respects to the control group (Chi square test, 90% vs. 5% for narcissism, 85% vs. 8% for sexual traumas, p < 0,001). Then, considering the overall Rorschach’s responses of thalassemic women, the prevalent characteristics may be summarized as follow: reduced number of responses, predominance of global responses, few responses of movement, many responses of anatomic parts of body, few responses determined by colour and hard control on the emotional life. The thalassemic patient faces this situation with similar defensive reactions that she usually exhibits relating to her own difficulties in life. Therefore, the Rorschach’s test and the clinical interview resulted very helpful to study deep psychological attitudes of the patients. The defensive and compensative value of somatic alterations against the latent risk of a disunion bent of ego would be confirmed by the existence of a phantasms-based forced life, able to create an excess of somatic phantasms of determined somatic images. These images may be documented by the mean of the inkblot pictures of the Rorschach’s test with an enormous number of anatomic interpretations, considering the representation of internal organs. The thalassemic patients have often red shock and refusal for the sixth inkblot picture. Aside sexual traumas, another evident characteristic shown by this study were narcissism as a fundamental personality trait; in fact the thalassemic women often see reflected mirror images.

These attitudes are expressed as omnipotent and oppositional behaviour. These patterns of challenging the nature hide an attempt to deny their own condition of illness and a defence from disease-related anxieties. Becoming pregnant is a way to feel as their peers; to feel normal and recovered. Therefore, pregnancy seems to have a therapeutic function. It doesn’t surprise that from the results of the Rorschach’s test arise narcissistic aspects and sexual traumas as principal personality traits.

During the first interview thalassemic women explained that they desired a baby because it was like a sort of challenge, a way to feel similar to normal women, to feel recovered by thalassemia and, in this way, overcome their main limit. On the contrary, for control group pregnancy is a way to be more women and to develop their own maternal role.

**Figure 2:**
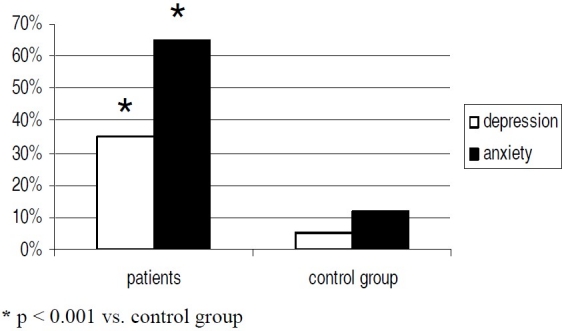
Score to SCL-90-R in thalassemic women and healthy group

**Figure 3:**
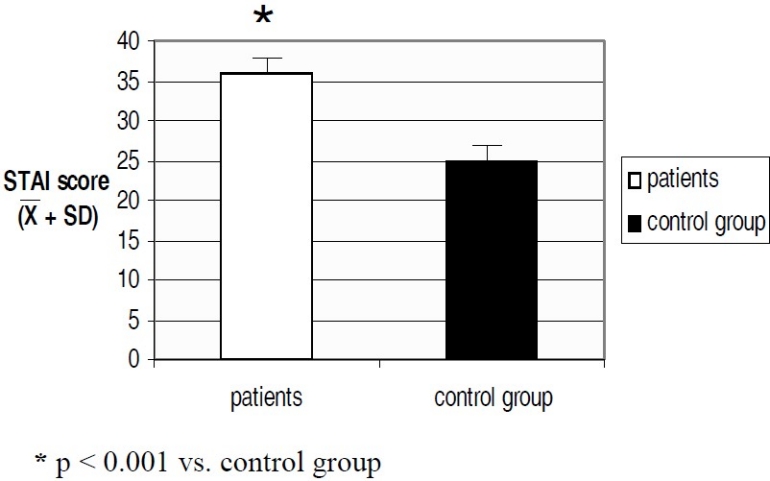
Score to STAI in thalassemic women and in healthy group

The percent of anxiety and depression observed with the SCL-90-R in study group was significantly higher than in control group (45% vs. 3%, p < 0.001, mean and SD values are 1.65 ± 0.15 vs. 0.43 ± 0.18 for anxiety; 55% vs. 12%, p < 0.001, mean and SD values are 1.76 ± 0.18 vs. 0.85 ± 0.25 for depression,Figure 2). Moreover the score observed with the STAI shows that the trait of anxiety differed significantly (87% vs. 42%, p < 0.05, mean and SD values are 33 ± 0.8 vs. 22 ± 0.2, Figure 3) between thalassemic pregnant women and the control group, even though the score values aren’t pathologic in neither group. The Chi square test and the Student’s t test adjusted with a correction factor was used, as appropriate. The score from STAI does not lead to a pathological anxiety condition but to an anxious personality side. The latter is a permanent condition of the personality while the former is transitory.

## Discussion

The thalassemic women personality seems to be marked by anxiety and depression, in addition to narcissistic features and sexual trauma. Studies on this topic put in evidence the same psychopathologic disorders, without highlighting the patients’ personality structure.^17^,^25^–^29^

The difficulties that thalassemic women encounter in order to become pregnant and the emotional burdens related to the many failed attempts of insemination and to the constant need of medical assistance^30^,^31^ made us wander what pushes these women to try to have a baby.^32^ Anyway, the value of a good reproductive and sexual life is increasingly acknowledged by the population, aside from the presence or absence of chronic diseases. Traditionally, adults with chronic illnesses have been considered asexual.^26^ Yet it is also acknowledged that reproductive health and sexuality are a major step in development through adolescence into adult life; influencing identity, self-esteem, social roles, and family planning. Young adults with thalassemia face the common sexual and reproductive issues, but they also have to deal with specific complications related to their disease and its treatment. These complications essentially arise from the chronic nature of the condition, the complexity of its treatment, and the prevalence of hypogonadotrophic hypogonadism.^7^,^33^,^34^ For this reason, thalassemic patients who decide to bear a child have to undergo many insemination attempts: in the analyzed sample the average number of attempts was 5.

## Conclusions

The multidisciplinary approach to treatment in patients suffering of thalassemia involves many professionals from different fields and encourages patients to take responsibility for their health and to acknowledge the holistic needs which they have as individuals. Although the present study provides some insights into the reproductive health experience of persons with homozygous thalassemia, further works are required. Areas of concern are education and employment experiences and aspirations, relationship experiences, and management of the patient’s fears and anxieties. Further studies are needed to provide substantial direction for future health care workers who have to deal with counselling such patients. To study pregnancy effects on thalassemic women and correlate the psychological status with neuroendocrine parameters will be the purpose of the future investigations. According to the recent discoveries of psychoneuroendocrinoimmunology (PNEI) showing the relation between psychological status and biological function mediated by the immune system, the evaluation of the psychological life will have to be included within the regular clinical investigations on thalassemic pregnant women.

The results of this first investigation could suggest that the therapeutic program which thalassemic women undergo to become pregnant should be integrated with a regular psychological therapy. Moreover the evaluation of the effects of pregnancy on the thalassemic disease will be the aim of future psychological investigations. The limit of this study is to analyze just thalassemic women because it doesn’t consider other pathologies. So the results can’t be extended to other pathologies different from thalassemic. Moreover, the low number of patients did not allow to draw defined conclusions. So, further studies will be required to establish the relation between pregnancy and psychological profile in thalassemic patients.

At the moment there are no studies evaluating psychologically aspects of pregnancy in thalassemic patients, since till few years ago their life expectance was very short. Previous studies just demonstrated the difficulty in pregnancy management in these women but didn’t consider the psychological status.

According to this study’s goals it can be affirmed that pregnancy in thalassemic patients often has a therapeutic value; it is perceived as a way to heal deep psychical wounds associated to experiences of distress and diversity from other women. Although these women suffer from anxiety and depression more frequently than healthy women do (see the SCL-90-R results), the key issue is that they undergo a hard path filled with emotional and physical burdens in order to be pregnant just to fix such aspects of themselves that they perceive unhealthy and eventually feel healthy and like other women. Furthermore, STAI shows that anxiety noticed in thalassemic women is really a point of their personality, as previous studies have already demonstrated. Therefore at the base of their personality there are clear narcissistic traits and sexual traumas.

## References

[1] Morelli M, Noia R, Zullo F, Corea D, Arduino B, Piccione F (2000). Pregnancy management in women with thalas-semia. Minerva Ginecol.

[2] Jensen CE, Tuck SM, Wonke B (1995). Fertility in beta thalassemia major: a report of 16 pregnancies, preconceptual evaluation and review of the literature. Br J Obstet Gynaecol.

[3] Palagiano A, Pace L (2001). Pregnancy in women with thalassemia. Minerva Ginecol.

[4] Hall MH (1974). Blood and neoplastic diseases. Pregnancy anaemia. Br Med J.

[5] Nathan DG (2005). Thalassemia: the continued challenge. Ann N Y Acad Sci.

[6] Maggio A, Maggio A, Hoffbrand AV (2004). Haemoglobinopathies today: from therapy to prevention. Clinical aspects and therapy of hemoglobinopathies.

[7] Skordis N, Christou S, Koliou M, Pavlides N, Angastiniotis M (1998). Fertility in female patients with thalassemia. J Pediatr Endocrinol Metab.

[8] Tuck SM (2005). Fertility and pregnancy in thalassemia major. Ann N Y Acad Sci.

[9] Najdecki R, Georgiou I, Lolis D (1998). The thalassemia syndromes and pregnancy, molecular basis, clinical aspects, prenatal diagnosis. Ginekol Pol.

[10] Surbek D, Koller A, Pavic N (1996). Successful twin pregnancy in homozygous beta-thalassemia after ovulation induction with growth hormone and gonadotropins. Fertil Steril.

[11] Danesi L, Scacchi M, Miragoli AM, Pincelli AI, Dubini A, Maiolo AT (1994). Induction of follicle maturation and ovulation by gonadotropin administration in women with beta-thalassemia. Eur J Endocrinol.

[12] Singh N, Deka D, Dadhwal V, Gupta N, Mittal S (2008). Optimizing antenatal care and delivery in thalassemic mothers: single center experience. Arch Gynecol Obstet.

[13] Toumba M, Kanaris C, Simamonian K, Skordis N (2008). Outcome and management of pregnancy in women with thalassemia in Cyprus. East Mediterr Health J.

[14] Pafumi C, Farina M, Pernicone G, Bandiera S, Russo A, Mangiafico L (2000). At term pregnancies in transfusion-dependent beta-thalassemic women. Clin Exp Obstet Gynecol.

[15] Pafumi C, Zizza G, Caruso S, Todaro AM, Pernicone G, Bandiera S (2000). Pregnancy outcome of a transfusion-dependent thalassemic woman. Ann Hematol.

[16] Karagiorga-Lagana M (1998). Fertility in thalassemia: the Greek experience. J Pediatr Endocrinol Metab.

[17] Messina G, Colombo E, Cassinerio E, Ferri F, Curti R, Altamura C (2008). Psychosocial aspects and psychiatric disorders in young adult with thalassemia major. Intern Emerg Med.

[18] Savona-Ventura C, Bonello F (1994). Beta-thalassemia syndromes and pregnancy. Obstet Gynecol Surv.

[19] Rorschach H (1941). Psychodiagnostics.

[20] Hassett AL, Radvanski DC, Buyske S, Savage SV, Sigal LH (2009). Psychiatric comorbidity and other psychological factors in patients with “chronic lyme disease”. Am J Med.

[21] Melloh M, Elfering A, Egli Presland C, Roeder C, Barz T, Rolli Salathe C (2009). Identification of prognostic factors for chronicity in patients with low back pain: a review of screening instrument. International Orthopaedics.

[22] Derogatis LR (1992). BSI: administration, scoring and procedures manual for the Brief Symptom Inventory II.

[23] Gorsuch RL, Lushene R, Spielberger CD, Vagg PR (1983). Manual for State-Trait Anxiety Inventory (Form Y).

[24] Spielberger CD, Reheiser EC, Ritterband LM, Sydeman SJ, Unger KK, Butcher JN (1995). Assessment of emotional states and personality traits: measuring psychological vital signs. Clinical personality assessment: practical approaches.

[25] Aessopos A, Karabatsos F, Farmakis D, Katsantoni A, Hatziliami A, Youssef J (1999). Pregnancy in patients with well-treated beta-thalassemia: outcome for mothers and newborn infants. Am J Obstet Gynecol.

[26] Kumar RM, Khuranna A (1998). Pregnancy outcome in women with beta-thalassemia major and HIV infection. Eur J Obstet Gynecol Reprod Biol.

[27] Goldbeck L, Baving A, Kohne E (2000). Psychosocial aspects of beta-thalassemia: distress, coping and adherence. Klin Padiatr.

[28] Ratip S, Modell B (1996). Psychological and sociological aspects of the Thalassemias. Semin Haematol.

[29] Ratip S, Skuse D, Porter J, Wonke B, Yardumian A, Modell B (1995). Psychosocial and clinical burden of Thalassemia intermedia and its implications for parental diagnosis. Arch Dis Chil.

[30] Aydinok Y, Erermis S, Bukusoglu N, Yilmaz D, Solak U (2005). Psychosocial implications of Thalassemia major. Pediatr Int.

[31] Ghanizadeh A, Khajavian S, Ashkani H (2006). Prevalence of psychiatric disorders, depression, and suicidal behaviour in child and adolescent with thalassemia major. J Pediatr Hematol Oncol.

[32] Butwick A, Findley I, Wonke B (2005). Management of pregnancy in a patient with beta thalassemia major. Int J Obstet Anesth.

[33] Insiripong S, Prabriputaloong S, Wisanuyothin N (2009). Thalassemic mothers and their babies. Southeast Asian J Trop Med Public Health.

[34] Horsmans Y, Karayiannis P, Christophe JL, Pickering JM, Debauche C, Cornu C (1999). Severe exacerbation of liver disease during pregnancy in a thalassemic GBV-C/HGV-positive patient and neonatal hepatitis in offspring. J Med Virol.

